# Pediatric meningiomas in The Netherlands 1974–2010: a descriptive epidemiological case study

**DOI:** 10.1007/s00381-012-1759-z

**Published:** 2012-04-29

**Authors:** Nikki B. Thuijs, Bernard M. J. Uitdehaag, Willem J. R. Van Ouwerkerk, Paul van der Valk, W. Peter Vandertop, Saskia M. Peerdeman

**Affiliations:** 1 Department of Epidemiology and Biostatistics, VU University Medical Center, PO Box 7057, 1007 MB Amsterdam, The Netherlands; 2 Department of Pathology, VU University Medical Center, PO Box 7057, 1007 MB Amsterdam, The Netherlands; 3 Meningioma Group Amsterdam, VU University Medical Center, PO Box 7057, 1007 MB Amsterdam, The Netherlands; 4 Neurosurgical Center Amsterdam, VU University Medical Center, PO Box 7057, 1007 MB Amsterdam, The Netherlands

**Keywords:** Meningioma, Childhood, Central nervous system, Brain neoplasm, Epidemiology

## Abstract

**Objective:**

The purpose of this study was to review the epidemiology and the clinical, radiological, pathological, and follow-up data of all surgically treated pediatric meningiomas during the last 35 years in The Netherlands.

**Methods:**

Patients were identified in the Pathological and Anatomical Nationwide Computerized Archive database, the nationwide network and registry of histopathology and cytopathology in The Netherlands. Pediatric patients of 18 years or younger at first operation in 1974–2009 with the diagnosis meningioma were included. Clinical records, follow-up data, radiological findings, operative reports, and pathological examinations were reviewed.

**Results:**

In total, 72 patients (39 boys) were identified. The incidence of operated meningiomas in the Dutch pediatric population is 1:1,767,715 children per year. Median age at diagnosis was 13 years (range 0–18 years). Raised intracranial pressure and seizures were the most frequent signs at presentation. Thirteen (18 %) patients had neurofibromatosis type 2 (NF2). Fifty-three (74 %) patients had a meningioma World Health Organization grade I. Total resection was achieved in 35 of 64 patients. Fifteen patients received radiotherapy postoperatively. Mean follow-up was 4.8 years (range 0–27.8 years). Three patients died as a direct result of their meningioma within 3 years. Four patients with NF2 died as a result of multiple tumors. Nineteen patients had disease progression, requiring additional treatment.

**Conclusion:**

Meningiomas are extremely rare in the pediatric population; 25 % of all described meningiomas show biological aggressive behavior in terms of disease progression, requiring additional treatment. The 5-year survival is 83.9 %, suggesting that the biological behavior of pediatric menigiomas is more aggressive than that of its adult counterparts.

## Introduction

Meningiomas account for about one-third of all primary central nervous system tumors and occur primarily in adults. In children, however, meningiomas are rare, accounting for less than 3 % of pediatric brain tumors, and therefore, only small, retrospective, single-institution series of pediatric meningiomas have been published. Only very few studies have analyzed the epidemiology and biological differences with the adult counterparts, showing quite different characteristics compared to adults.

The revised World Health Organization (WHO) criteria, with the prognostic significance of newer morphological variants (i.e., rhabdoid and chordoid tumors), now provide well-established guidelines for evaluating meningiomas in the adult population, but little is known about the frequency and prognostic importance of childhood cases. Some authors state that pediatric meningiomas are more aggressive than their adult counterparts; however, there is no consensus. A major problem in pediatric meningiomas is the discordance that arises between the histological appearance of the tumor and its biological behavior.

Using the nationwide network and registry of histopathology and cytopathology in The Netherlands, Pathological and Anatomical Nationwide Computerized Archive (PALGA), we performed an epidemiological analysis of childhood meningiomas in order to establish a better understanding of the behavior of these tumors in contrast to those in adults.

## Materials and methods

All patients were identified by searching the PALGA database (www.palga.nl), the nationwide network and registry of histopathology and cytopathology in The Netherlands, after appropriate institutional medical ethics committee approval was obtained. The search was conducted from January 1974 (first CT scan available in The Netherlands) to January 2010. All meningiomas (intracranial, intraspinal, and meningiomas outside the central nervous system (CNS)) in patients 18 years or younger at the time of histological diagnosis were included. Fibrous histiocytomas, meningeal melanomas, and angioblastic meningeal tumors were excluded. Histological subtypes were classified on the basis of WHO criteria valid at the time of presentation [27]. Meningosarcomas were classified as WHO grade III [4, 28].

Subsequently, all Dutch neurosurgical centers were visited to retrospectively review in detail the clinical records, radiological findings, operative reports, and pathological examinations and to cross check this with the PALGA database description. Data on gender, age at diagnosis, medical history, family history, comorbidity, symptoms, imaging, tumor location, tumor resection grade, histology, additional treatment, and follow-up were retrieved. Recurrence was defined as the appearance of recurrent tumor tissue at the same location after gross total resection (GTR) or the growth of residual tumor tissue after subtotal resection (STR). Time to recurrence was defined as the time from GTR to radiological reappearance of a new tumor. For the patients with known follow-up, survival probabilities were calculated according to the Kaplan–Meier method and were measured from the date of diagnosis until the date of last follow-up or death. Estimation of the Dutch pediatric population was made on the basis of the number of persons of 18 years or younger in 1992, the median of our time span. A two-sided probability level of 0.05 was chosen for statistical significance. Statistical analysis was performed using SPSS for Windows (version 17.0; SPSS, Inc., Chicago, IL).

## Results

From 1974 to 2010, one hundred and seventeen entries of meningioma histology in patients aged 18 years or younger were identified in the PALGA database. Forty-five cases were excluded because either the histological diagnosis was a duplicate (two PALGA registries for the same tumor in the same patient) or the original histological diagnosis was changed after immediate revision. The final study group consisted of 72 patients (Table 1).

**Table 1:** Disease progression and mortality rates among different variables

Variable	No. of cases	Mean follow-up (years)	Disease progression *n* (%)	Mortality *n* (%)
Age (years)				
0–5	13	4.7	5 (38)	2 (15)
6–11	19	6.0	7 (37)	0
12–18	40	4.3	7 (18)	5 (13)
Gender				
Male	39	4.9	10 (26)	4 (10)
Female	33	4.7	9 (27)	3 (9)
Location				
Supratentorial	38	5.0	11 (29)	3 (8)
Infratentorial	13	5.5	4 (31)	4 (31)
Spine	12	4.4	2 (15)	0
Orbit	4	1.9	2 (50)	0
Cutaneous	5	NA	NA	0
NF2				
Yes	13	7.8	4 (31)	4 (31)
No	59	4.1	15 (25)	3 (51)
RTH history				
Yes	4	4.8	2 (50)	0
No	68	5.3	17 (25)	7 (10)
WHO grade				
I	53	4.8	15 (28)	4 (8)
II	13	2.7	3 (23)	1 (8)
III	6	9.9	1 (17)	2 (33)
Procedure^a^				
GTR	35	4.7	6 (17)	0
STR	29	4.0	13 (45)	4 (14)
RGND	8	5.3	0	3 (38)
Total	72	4.9	19 (26)	7 (10)

*NA* not applicable, *NF2* neurofibromatosis type 2, *RTH* radiotherapy, *WHO* World Health Organization, *GTR* gross total resection, *STR* subtotal resection, *RGND* resection grade not described

^a^At first operation

Seventy-two pediatric meningiomas were operated upon in 35 years in The Netherlands. This equates to two operated meningiomas in patients 18 years or younger a year. As 1992 is the median of our time span, a year in which the pediatric Dutch population consisted of 3,535,430 people [24], the incidence is 1 per 1,767,715 persons younger than 19 years.

### Clinical characteristics

Of the 72 patients, 39 were male, for a male/female ratio of 1.18. The mean age at diagnosis was 11.8 years (range 0.3–18.8) with a median age of 12.7 years (interquartile range eight years). Age did not differ significantly between the two gender groups.

Thirteen (18 %) out of the 72 patients had neurofibromatosis (NF). The NF group was similar to the entire group with regard to age, resection grade, and location. However, the NF group had a female predominance (*n* = 8, not significant), more often multiple meningiomas at presentation (*n* = 7), and shorter duration of symptoms: mean 5.8 versus 11.7 months (not significant). No WHO grade III meningiomas and two out of 14 WHO grade II meningiomas were present in this NF group.

Five patients had meningiomas outside the CNS, located extracranially in the cranial subcutaneous tissue. Four patients in this study had previously received radiation of the central nervous system for medulloblastoma, astrocytoma, ependymoma, and Hodgkin’s disease. These four patients had a WHO grade I meningioma. Two of these four meningiomas received additional radiotherapy after surgery. One patient suffered neurofibromatosis type 2 (NF2) and had two meningiomas at first presentation. One of these meningiomas showed recurrence and was operated upon 3 years later. Growth of this tumor and development of two new tumors occurred again 3 years later. The mean latency period between radiotherapy and the diagnosis of meningioma was 8.5 years (range 4–13).

### Presenting symptoms

Median duration of symptoms of the CNS tumors was 5 months (average 11, range 0–72). Although presenting symptoms depended on tumor location, meningiomas most often (36 %) presented with signs and/or symptoms of raised intracranial pressure (headache, vomiting, papilledema; *n* = 26) or epilepsy (28 %).

Three out of 13 patients with NF2 had stigmata of neurofibromatosis (café-au-lait spots and Lisch nodules). Three patients had a cranial deformity as a result of tumor invasion of the skull. All five patients with a cutaneous meningioma presented with a lump on the head, which was present since birth.

### Anatomical distribution

The location of tumors at first presentation is presented in Table 1. The majority of tumors was located at the convexity (*n* = 27). None of the five patients with subcutaneous meningiomas showed intracranial invasion. Eight patients had multiple central nervous system tumors at first presentation, seven of whom had NF2.

### Histology

The distribution of WHO grade is presented in Table 1. Meningotheliomatous meningioma was the most common histological subtype (*n* = 15). Of the WHO grade II meningiomas, clear cell and atypical subtypes were the most common (*n* = 5). Of the six WHO grade III tumors, two were meningosarcomas, two anaplastic, one hemangioperycital, and one of a rhabdoid subtype. WHO grade II meningiomas appeared to occur more in adolescents than in the younger patients (not significant).

### Additional treatment

**Fig. 1:**
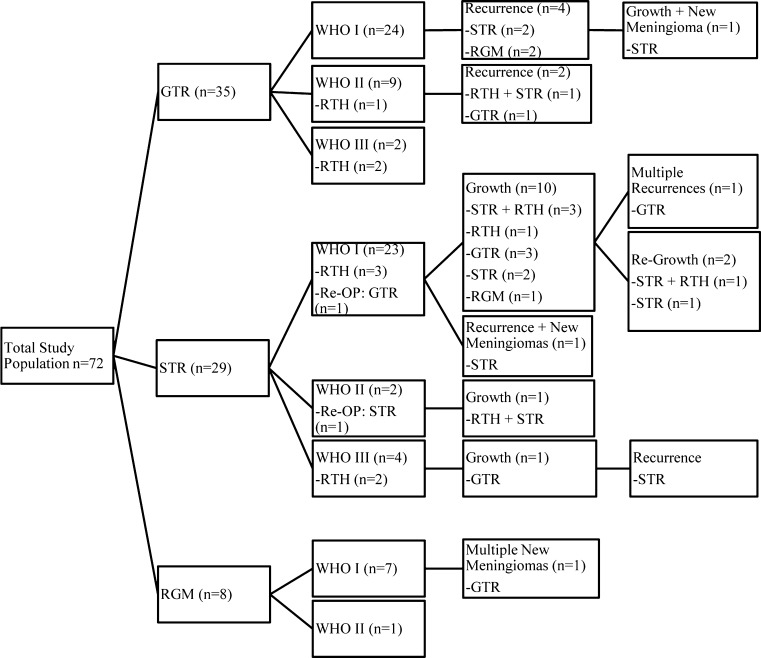
*GTR* gross total resection, *STR* subtotal resection, *OP* operation, *RGM* resection grade missing, *RTH* radiotherapy, *WHO* World Health Organization

At first surgery, GTR was accomplished in 35 patients and STR in 29 patients. Simple decompression was performed in six patients (Fig. 1). Reasons for simple decompression were either an inoperable tumor or a planned biopsy. GTR and STR numbers were achieved in equal percentages among the various age groups.

Additional surgery within 30 days after first surgery was performed in five patients. Reasons were dural reconstruction after cerebral spinal fluid leakage, or placement or removal of a shunt due to hydrocephalus.

Fifteen patients (11 males) received radiotherapy (RTH) to the central nervous system postoperatively (Fig. 1). Mean dose given was 42.75 cGy (range 13–60). Additional RTH followed an incomplete resection in ten patients (WHO I *n* = 7, WHO II *n* = 1, WHO III *n* = 2) and complete resection in five (WHO I *n* = 1, WHO II *n* = 2, WHO III *n* = 2). Reasons for RTH after GTR were recurrence or atypical or malignant tumor tissue. Three patients received RTH twice. Mean interval between two sessions of RTH was 5 years (range 1–10 years).

### Follow-up

**Fig. 2:**
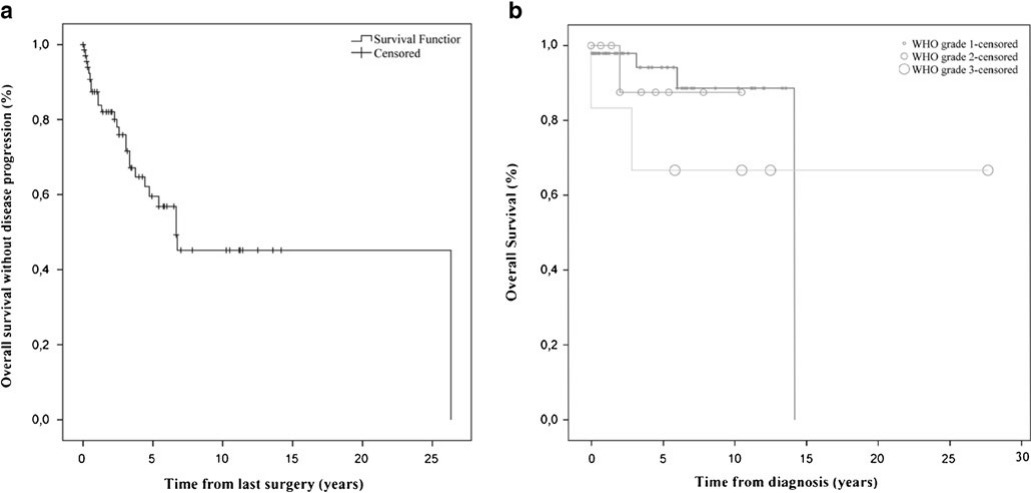
*n* = 67 (excluding cutaneous meningiomas). **a** Graph demonstrating disease progression. **b** Graph showing overall survival by WHO grade

Mean follow-up was 4.8 years (0–27.8 years, Fig. 2a). Seventy-two patients had 77 meningiomas at first presentation, of which 76 were removed at first surgery. In total, 72 patients had 93 meningiomas, which were operated upon 105 times.

Of the 105 meningioma removals, 46 meningiomas were resected totally; 43 were resected subtotally; and in 16 meningiomas, resection grade was not described. Of the 46 meningiomas resected completely, 12 recurred in nine patients within a mean period of 5.6 years (range 1–26). Of the 43 meningiomas resected subtotally, 14 showed significant growth that required reoperation and/or RTH within 1.5 years (range 0–4).

Time to reoperation was 3.9 years (range 0–26). A flow chart of treatment sequelae is shown in Fig. 1. Four patients developed a second meningioma or a neuroectodermal tumor of a variable histological type, e.g., schwannoma,or ependymoma.

Seven of the 72 patients are known to have died, three as a direct result of their meningioma and four patients with NF2 died as a result of multiple tumors. One 13-year-old patient had pulmonary metastasis of his WHO grade III meningioma. Mean interval time between diagnosis and death was 4 years (range 0–14).

The 1- and 5-year overall survival rate of the CNS meningiomas was 96.0 % (*n* = 50) and 83.9 % (*n* = 31), respectively. The progression-free survival after 1 and 5 years was 84.3 % (*n* = 51) and 55.6 % (*n* = 36), respectively (Fig. 2b).

## Discussion

This is the first study describing all cases of operated pediatric meningiomas in one entire country, providing an excellent opportunity to determine an accurate incidence rate. It covers all registered pediatric meningioma data from the past 35 years, and more importantly, it is the third largest series on pediatric meningioma [13, 21].

With an incidence of 1:1,770,000 in the pediatric population in The Netherlands, meningiomas contribute to only a small proportion of all pediatric tumors. This very low incidence of childhood meningiomas has been confirmed in many literature studies [15]. We used 18 years as the upper age limit. In other papers, the age range varies widely in the inclusion criteria.

The pediatric literature is based on extensive time periods during which imaging facilities, pathological criteria, and surgical advances have led to shifts in multiple variables, which makes comparison of results difficult. In our series, there were 19 atypical and malignant meningiomas (26 %), compared with 2–38 % cited in the literature [5]. In the the adult population, this percentage is lower: about 10 % of all meningiomas [1, 6, 8, 22].

A difference between adult and pediatric meningiomas is the higher percentage of patients with disease progression. This might even be a considerable underestimation, since many patients were lost to follow-up. This higher recurrence rate reflects a more aggressive biological behavior of pediatric meningiomas.

Kotecha et al. have published a meta-analysis of individual pediatric patient data in 2012. They found an overall survival after 5 years of 95.0 % in totally resected pediatric meningiomas. This survival decreases to 77.3 % in subtotally resected meningiomas. Notably, in adults, the overall survival after 5 years is higher compared with the 84 % in our series and 82 % in the serie of Kotecha et al. [13].

As in adults, the extent of resection is an important prognostic factor as recurrence-free rates improve significantly with an increasing extent of removal [13]. In our series, however, the 5-year survival rates are clearly lower (84 %) than those reported in adults (Table 2).

**Table 2:** Adult and pediatric meningioma characteristics

Variable	Adult	Pediatric
Prevalence	97.5/100,000 [26]	0.06/100,000^a^
Male to female ratio	2:1 [26]	1.18:1^a^
Location		
Most common	Convexity	Convexity^a^
Infratentorial	8 %	20 %
Intraventricular	Rare	Frequent 11 %
Dural attachment	Frequent	Rare
Histology subtype		
Most common	Meningothelial	Meningothelial^a^
WHO grades II and III	8 % [14]	19–32 %
5-year survival		
Overall	93 % [25]	84 %^a^
WHO I	96 %	93 %
WHO II	78^9^–95 % [19]	87 %
WHO III	44^9^–64 % [19]	72 %
5-year RFS		
WHO I	88–100 % [18]	81 %
WHO II	48–73 % [9]	69 %
WHO III	16–61 % [9]	41 %

*RFS* relapse-free survival, *WHO* World Health Organization

^a^Present study

Kotecha et al. demonstrated a key prognostic factor for progression-free survival. Children who undergo initial GTR have a significantly better progression-free survival than those with an initial STR [13], as in our study. This also accounts that overall survival is also related to location of the tumor [14, 17].

Most pediatric CNS tumors have a worse prognosis under the age of 6 years. Worse overall survival in this age group might be explained by congenital tumor development, which often results in tumors that are more aggressive in their biological behavior [13].

In our present series, 18 % had neurofibromatosis, which fits within the range reported in other series [2, 7, 10, 11]. In our study, NF2-associated meningiomas did not differ significantly from sporadic pediatric tumors with regard to clinicopathological features, but they did have a higher frequency of multiplicity [12].

What is extraordinary in this series is the number of meningiomas located outside the CNS. Five out of the 72 meningiomas were seen in the cranial subcutaneous tissue. Meningiomas of the skin are the rarest of all extracranial meningiomas and have been reported in the scalp, the forehead, and the paravertebral region, where its occurrence is exceptional [23].

Meningiomas are recognized as the most common long-term complication following radiotherapy to the CNS [3, 20]. Typically, the latent period for the presentation of such tumors is 15 to 20 years; however, they may occur as early as 4 years after radiation treatment [16]. In our study, four patients developed meningiomas within 13 years after radiation.

The follow-up time of our study is short (mean 4.9 years), taking into account that the entire study covers 35 years. Only 31 patients have a follow-up time of 5 years, due to the retrospective character of this study. Therefore, it is possible that the recurrence rate is underestimated and biological behavior is even worse than described.

In conclusion, pediatric meningiomas are very rare compared to their counterparts in adults. With an incidence of approximately 1:1.8 million operated patients in the pediatric population in The Netherlands, meningiomas contribute to only a small proportion of all pediatric tumors. Pediatric meningiomas have a more aggressive biological behavior compared to those of the adult population with a high proportion of patients showing disease progression after surgery. Moreover, the 5-year survival rate was only 83.9 %, and this is probably even an underestimation.

Despite the retrospective character of this study and its limitations, one can state that biological behavior of pediatric meningiomas is more aggressive than of the adult counterparts. Further research into biomarkers might give insights into possible explanations for these differences in behavior.

## References

[1] Alvarez F, Roda JM, Perez RM, Morales C, Sarmiento MA, Blazquez MG (1987). Malignant and atypical meningiomas: a reappraisal of clinical, histological, and computed tomographic features. Neurosurgery.

[2] Baumgartner JE, Sorenson JM (1996). Meningioma in the pediatric population. J Neurooncol.

[3] Brassesco MS, Valera ET, Neder L, Castro-Gamero AM, de Oliveira FM, Santos AC, Scrideli CA, Oliveira RS, Machado HR, Tone LG (2009). Childhood radiation-associated atypical meningioma with novel complex rearrangements involving chromosomes 1 and 12. Neuropathology.

[4] Buttner A, Pfluger T, Weis S (2001). Primary meningeal sarcomas in two children. J Neurooncol.

[5] Caroli E, Russillo M, Ferrante L (2006). Intracranial meningiomas in children: report of 27 new cases and critical analysis of 440 cases reported in the literature. J Child Neurol.

[6] Davidson GS, Hope JK (1989). Meningeal tumors of childhood. Cancer.

[7] Deen HG, Scheithauer BW, Ebersold MJ (1982). Clinical and pathological study of meningiomas of the first two decades of life. J Neurosurg.

[8] Drake JM, Hendrick EB, Becker LE, Chuang SH, Hoffman HJ, Humphreys RP (1985). Intracranial meningiomas in children. Pediatr Neurosci.

[9] Durand A, Labrousse F, Jouvet A, Bauchet L, Kalamaridès M, Menei P, Deruty R, Moreau JJ, Fèvre-Montange M, Guyotat J (2009). WHO grade II and III meningiomas: a study of prognostic factors. J Neurooncol.

[10] Erdincler P, Lena G, Sarioglu AC, Kuday C, Choux M (1998). Intracranial meningiomas in children: review of 29 cases. Surg Neurol.

[11] Germano IM, Edwards MS, Davis RL, Schiffer D (1994). Intracranial meningiomas of the first two decades of life. J Neurosurg.

[12] Kotecha RS, Junckerstorff RC, Lee S, Cole CH, Gottardo NG (2011) Pediatric meningioma: current approaches and future direction. J Neurooncol 10.1007/s11060-010-0503-3 21203895

[13] Kotecha RS, Pascoe EM, Rushing EJ, Cole CH, Gottardo NG (2011). Meningiomas in children and adolescents: a meta-analysis of individual patient data. Lancet Oncol.

[14] Mahmood A, Caccamo DV, Tomecek FJ, Malik GM (1993). Atypical and malignant meningiomas: a clinicopathological review. Neurosurgery.

[15] Menon G, Nair S, Sudhir J, Rao BR, Mathew A, Bahuleyan B (2009). Childhood and adolescent meningiomas: a report of 38 cases and review of literature. Acta Neurochir (Wien).

[16] Moss SD, Rockswold GL, Chou SN, Yock D, Berger MS (1988). Radiation-induced meningiomas in pediatric patients. Neurosurgery.

[17] Numaguchi Y, Hoffman JC, O’Brien MS, Fukui M, Matsuura K, Kitamura K (1978). Meningiomas in childhood and adolescence. Neurol Med Chir (Tokyo).

[18] Onodera S, Aoyama H, Katoh N, Taguchi H, Yasuda K, Yoshida D, Surtherland K, Suzuki R, Ishikawa M, Gerard B, Terasaka S, Shirato H (2011). Long-term outcomes of fractionated stereotactic radiotherapy for intracranial skull base benign meningiomas in single institution. Jpn J Clin Oncol.

[19] Palma L, Celli P, Franco C, Cervoni L, Cantore G (1997). Long-term prognosis for atypical and malignant meningiomas: a study of 71 surgical cases. Neurosurg Focus.

[20] Pettorini BL, Park YS, Caldarelli M, Massimi L, Tamburrini G, Di Rocco C (2008). Radiation-induced brain tumours after central nervous system irradiation in childhood: a review. Childs Nerv Syst.

[21] Rushing EJ, Olsen C, Mena H, Rueda ME, Lee YS, Keating RF, Packer RJ, Santi M (2005). Central nervous system meningiomas in the first two decades of life: a clinicopathological analysis of 87 patients. J Neurosurg.

[22] Scheithauer BW (1990). Tumors of the meninges: proposed modifications of the World Health Organization classification. Acta Neuropathol.

[23] Shuangshoti S, Panyathanya R (1973). Ectopic meningiomas. Arch Otolaryngol.

[24] Statistics Netherlands (2009) Population, key figures

[25] Talback M, Stenbeck M, Rosen M (2004). Up-to-date long-term survival of cancer patients: an evaluation of period analysis on Swedish Cancer Registry data. Eur J Cancer.

[26] Wiemels J, Wrensch M, Claus EB (2010). Epidemiology and etiology of meningioma. J Neurooncol.

[27] Zulch KJ (1981). Historical development of the classification of brain tumours and the new proposal of the World Health Organization (WHO). Neurosurg Rev.

[28] Zwartverwer FL, Kaplan AM, Hart MC, Hertel GA, Spataro J (1978). Meningeal sarcoma of the spinal cord in a newborn. Arch Neurol.

